# Altered white matter connectivity associated with visual hallucinations following occipital stroke

**DOI:** 10.1002/brb3.1010

**Published:** 2018-05-21

**Authors:** Sara A. Rafique, John R. Richards, Jennifer K. E. Steeves

**Affiliations:** ^1^ Department of Psychology Centre for Vision Research York University Toronto ON Canada; ^2^ Department of Emergency Medicine University of California, Davis, Medical Center Sacramento California

**Keywords:** diffusion tensor imaging, magnetic resonance imaging, stroke, vision loss, visual hallucinations, white matter

## Abstract

**Introduction:**

Visual hallucinations that arise following vision loss stem from aberrant functional activity in visual cortices and an imbalance of activity across associated cortical and subcortical networks subsequent to visual pathway damage. We sought to determine if structural changes in white matter connectivity play a role in cases of chronic visual hallucinations associated with visual cortical damage.

**Methods:**

We performed diffusion tensor imaging (DTI) and probabilistic fiber tractography to assess white matter connectivity in a patient suffering from continuous and disruptive phosphene (simple) visual hallucinations for more than 2 years following right occipital stroke. We compared these data to that of healthy age‐matched controls.

**Results:**

Probabilistic tractography to reconstruct white matter tracts suggests regeneration of terminal fibers of the ipsilesional optic radiations in the patient. However, arrangement of the converse reconstruction of these tracts, which were seeded from the ipsilesional visual cortex to the intrahemispheric lateral geniculate body, remained disrupted. We further observed compromised structural characteristics, and changes in diffusion (measured using diffusion tensor indices) of white matter tracts in the patient connecting the visual cortex with frontal and temporal regions, and also in interhemispheric connectivity between visual cortices.

**Conclusions:**

Cortical remapping and the disruption of communication between visual cortices and remote regions are consistent with our previous functional magnetic resonance imaging (fMRI) data showing imbalanced functional activity of the same regions in this patient (Rafique et al, 2016, Neurology, 87, 1493–1500). Long‐term adaptive and disruptive changes in white matter connectivity may account for the rare nature of cases presenting with chronic and continuous visual hallucinations.

## 1 INTRODUCTION

Visual hallucinations can occur as a secondary consequence of vision loss in individuals who are otherwise cognitively healthy. The type of hallucinatory image is related to the site of damage along the visual pathway, and/or the visual processing function of the implicated region(s). Lesions of the optic radiations, calcarine, and early visual cortices can result in simple (phosphene) hallucinations, whereas involvement of higher visual processing areas is associated with complex visual hallucinations (ffytche, 2008; Vaphiades, Celesia, & Brigell, 1996).

Previous functional magnetic resonance imaging (fMRI) studies have shown visual hallucinations following visual pathway damage are associated with disorganized functional activity in visual cortices across both hemispheres, and in interconnected cortical and subcortical networks (ffytche et al., 1998; Rafique, Richards, & Steeves, 2016). We sought to determine if structural changes in white matter connectivity are also present in cases of chronic visual hallucinations following visual pathway damage. Diffusion tensor imaging (DTI) and probabilistic fiber tractography enable noninvasive investigation of white matter structure. DTI quantifies the diffusion of water in white matter tracts, which is influenced by underlying microstructure and orientation of axonal fibers, and provides information about their integrity. Water diffusion can be characterized by diffusion tensor indices: fractional anisotropy (FA; directionality of diffusion parallel to fiber tracts), mean diffusivity (MD; directionally averaged measure of diffusion), axial diffusivity (AD; measure of diffusion parallel to axonal fibers), and radial diffusivity (RD; measure of diffusion perpendicular to axonal fibers). Fiber tracking algorithms use directions of greatest diffusion to estimate white matter fiber orientation (Ciccarelli, Catani, Johansen‐Berg, Clark, & Thompson, 2008). Probabilistic fiber tractography employs Bayesian modeling to infer these fiber directions and generate three‐dimensional depictions of white matter tracts (Behrens et al., 2003).

We investigated a patient experiencing chronic and continuous phosphene visual hallucinations following occipital stroke. In a previous study, the patient underwent fMRI and therapeutic treatment with repetitive transcranial magnetic stimulation (rTMS) to the lesion site to provide relief from hallucinations that were interfering with quality of life. fMRI showed aberrant functional activity in visual cortices, and an imbalance of activity across frontal, parietal, temporal, and cerebellar regions prior to rTMS treatment (Rafique et al., 2016). In this study, we investigated whether the previously documented aberrant functional activity and persistent nature of hallucinations in the patient (Rafique et al., 2016) were a reflection of compromised structural characteristics of white matter connectivity. We used DTI and probabilistic tractography to examine white matter connectivity between cortical regions implicated in our previous fMRI study. Moreover, in our previous fMRI study, we observed some restoration of functional activity in the patient following treatment with rTMS. However, this redistribution of functional activity in the patient did not completely match that of controls; and despite a significant reduction in intensity of visual hallucination with rTMS treatment, the hallucinations persisted, albeit to a lesser extent (Rafique et al., 2016). We further expected that this incomplete functional restoration and failure to fully suppress the hallucinations with rTMS would in part, if not wholly, be due to white matter connectivity changes. There are presently no cases in the literature that have investigated white matter in cases of visual hallucinations following visual pathway damage.

## 2 METHODS

The study was approved by York University’s Office of Research Ethics. All individuals gave informed written consent.

### 2.1 Patient and control participants

**Figure 1:**
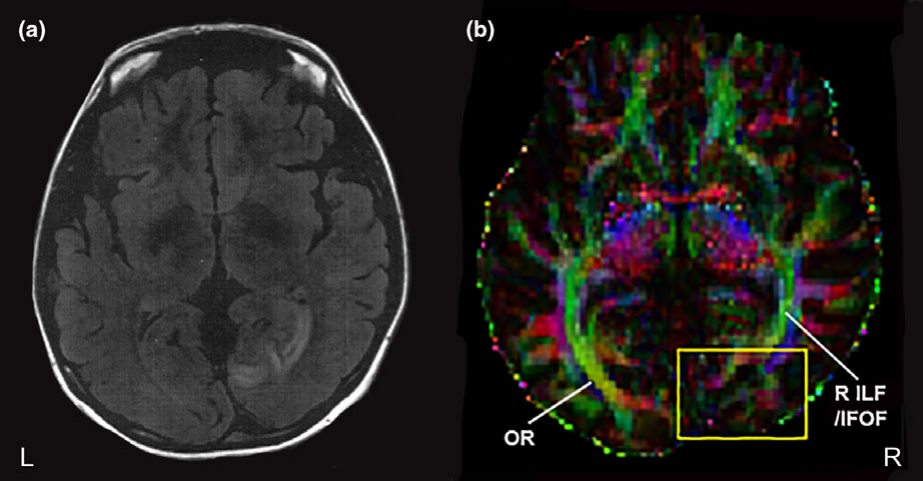
MR images for the patient. (a) T2‐weighted image shows right occipital ischemia at the time of stroke. (b) Diffusion tensor image of a color‐coded FA map for the patient. Reduced anisotropy is observed at the lesion site (yellow box), with disruption in diffusivity at the right (R) inferior longitudinal fasciculus (ILF)/inferior fronto‐occipital fasciculus (IFOF), which merge with the optic radiations (OR). Red represents fibers crossing left to right, green represents fibers in the posterior‐anterior direction, and blue represents fibers in the inferior‐superior direction. Average MNI coordinates of lesion site: *x* = 15, *y* = −68, *z* = 5. FA: fractional anisotropy

In brief, we evaluated a 31‐year‐old female experiencing persistent and disruptive phosphene visual hallucinations within an incomplete congruous left homonymous hemianopia following right subacute occipital stroke. At the time of initial investigation, occipital ischemia (Figure 1a) and calcarine artery vasospasm were observed. Electroencephalogram (EEG) recordings were normal, ruling out an epileptic origin for the visual hallucinations. We assessed the patient 2.5 years following stroke and onset of phosphene hallucinations, at which time the calcarine artery vasospasm and ischemia had resolved. The phosphene hallucinations consisted of moving and vibrating white/gray images resembling shattered glass, small‐medium mostly circular lights, and occasional lightning bolts and irregular zigzag lines. The patient history is described in more detail in our previous study (Rafique et al., 2016).

Seven age‐matched female control participants (mean age = 29 years) with no history of neurological disorders, and normal or corrected‐to‐normal vision (>0.2 logMAR) were recruited. All participants were right‐handed with no known contraindications to MRI. All participants had taken part in a previous fMRI study (Rafique et al., 2016).

### 2.2 Magnetic resonance imaging acquisition

Imaging was acquired with a 3‐Tesla Siemens Magnetom^®^ Tim Trio magnetic resonance scanner and a Siemens 32 channel high‐resolution brain array coil (Siemens, Erlanger, Germany). Whole‐brain diffusion‐weighted scanning, T1‐weighted, and T2‐weighted turbo spin echo fluid‐attenuated inversion recovery (FLAIR) imaging sequences were obtained. Full details on MRI acquisition are provided in Supporting Information.

### 2.3 Data analyses

All DTI analyses were performed using FSL’s DTI processing pipeline (FMRIB, Oxford, UK; http://www.fmrib.ox.ac.uk/fsl).

#### 2.3.1 Image processing and normalization

Diffusion images underwent preprocessing. T1‐weighted and diffusion images were registered to standard MNI space (MNI152 1 × 1 × 1 mm). Full details on image processing and normalization to standard space are provided in Supporting Information.

#### 2.3.2 Diffusion tensor indices

The diffusion tensor indices FA, MD, AD, and RD were extracted from eleven major white matter tracts of John Hopkins University (JHU) White‐Matter Tractography Atlas (Wakana et al., 2007) for each participant using Tract‐Based Spatial Statistics (TBSS; Smith et al., 2006). Diffusion tensor indices were extracted from the following bilateral tracts: anterior thalamic radiations, corticospinal tracts, cingulum (cingulate gyrus and hippocampus), inferior fronto‐occipital fasciculi, inferior longitudinal fasciculi, superior longitudinal fasciculi, temporal aspects of superior longitudinal fasciculi, and uncinate fasciculi. Diffusion tensor indices were also extracted from the forceps major and minor. Full details on obtaining diffusion tensor indices from white matter tracts are provided in Supporting Information. The extracted diffusion tensor indices underwent statistical analyses to compare the patient with controls using the modified independent one‐tailed *t* test for single case studies (Crawford & Garthwaite, 2002). We report significant differences in diffusion tensor indices (Table 1) at *p *<* *0.05, and trending significance at *p *<* *0.1.

**Table 1:** Diffusion tensor indices for major white matter tracts

White matter tracts	FA		MD (×10^−3^)		AD (×10^−3^)		RD (×10^−3^)	
	Controls	Patient	Controls	Patient	Controls	Patient	Controls	Patient
R ATR	0.508 ± 0.020	0.501	0.692 ± 0.017	0.693	1.102 ± 0.020	1.099	0.487 ± 0.020	0.490
L ATR	0.513 ± 0.014	0.484 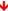	0.695 ± 0.020	0.689	1.108 ± 0.035	1.073	0.489 ± 0.016	0.497
R cCG	0.574 ± 0.035	0.625	0.671 ± 0.029	0.676	1.136 ± 0.055	1.221	0.438 ± 0.035	0.403
L cCG	0.607 ± 0.031	0.628	0.689 ± 0.026	0.688	1.221 ± 0.063	1.247	0.424 ± 0.029	0.409
R cH	0.588 ± 0.063	0.551	0.558 ± 0.058	0.637	0.928 ± 0.079	1.049	0.373 ± 0.061	0.432
L cH	0.588 ± 0.052	0.546	0.594 ± 0.051	0.593	1.014 ± 0.073	0.971	0.384 ± 0.054	0.404
R CT	0.626 ± 0.014	0.608	0.664 ± 0.018	0.670	1.197 ± 0.030	1.179	0.397 ± 0.018	0.415
L CT	0.633 ± 0.012	0.612 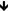	0.663 ± 0.022	0.673	1.202 ± 0.047	1.195	0.387 ± 0.009	0.412 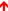
FMa	0.685 ± 0.022	0.675	0.718 ± 0.019	0.726	1.423 ± 0.025	1.410	0.366 ± 0.027	0.383
FMi	0.570 ± 0.024	0.564	0.718 ± 0.019	0.729	1.249 ± 0.023	1.261	0.452 ± 0.025	0.463
R IFOF	0.562 ± 0.030	0.530	0.726 ± 0.019	0.751	1.236 ± 0.020	1.240	0.471 ± 0.032	0.506
L IFOF	0.561 ± 0.022	0.548	0.750 ± 0.017	0.735	1.280 ± 0.018	1.240 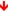	0.486 ± 0.023	0.482
R ILF	0.562 ± 0.028	0.517 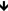	0.724 ± 0.017	0.737	1.229 ± 0.023	1.240 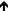	0.471 ± 0.027	0.509
L ILF	0.549 ± 0.020	0.555	0.748 ± 0.019	0.727	1.265 ± 0.023	1.240	0.490 ± 0.023	0.471
R SLF	0.535 ± 0.019	0.497 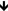	0.686 ± 0.020	0.689	1.118 ± 0.027	1.080	0.468 ± 0.018	0.494
L SLF	0.516 ± 0.013	0.490 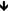	0.716 ± 0.017	0.714	1.146 ± 0.017	1.108 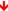	0.501 ± 0.020	0.518
R tSLF	0.586 ± 0.025	0.539 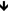	0.672 ± 0.021	0.683	1.149 ± 0.031	1.116	0.433 ± 0.026	0.467
L tSLF	0.557 ± 0.017	0.532	0.716 ± 0.017	0.719	1.193 ± 0.016	1.159 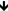	0.478 ± 0.021	0.499
R UF	0.518 ± 0.036	0.498	0.736 ± 0.027	0.734	1.206 ± 0.044	1.165	0.502 ± 0.037	0.519
L UF	0.514 ± 0.029	0.487	0.736 ± 0.018	0.730	1.192 ± 0.029	1.172	0.507 ± 0.027	0.508

*Notes*

AD: axial diffusivity; ATR: anterior thalamic radiation; cCG: cingulum (cingulate gyrus); cH: cingulum (hippocampus); CT: corticospinal tract; FA, fractional anisotropy; FMa: forceps major; FMi: forceps minor; IFOF: inferior fronto‐occipital fasciculus; ILF: inferior longitudinal fasciculus; L: left; MD: mean diffusivity; R: right; RD: radial diffusivity; SLF: superior longitudinal fasciculus; tSLF: temporal aspect of superior longitudinal fasciculus; UF: uncinate fasciculus.

The columns list (from left to right) the major white matter tracts and their associated diffusion tensor indices for controls and the patient. Data are presented as mean (±*SD*) control and single patient values.

Arrows indicate significant increase/decrease in patient’s diffusion tensor index relative to controls (black arrows represent *p *<* *0.1; red arrows represent *p *<* *0.05).

#### 2.3.3 Probabilistic fiber tractography

Our main interests were tracts connected with the visual cortex (primary and association cortices), consistent with the location of the patient’s lesion site. Tracts of interest were generated to cortical regions of interest based on our previous fMRI findings, which showed functional differences in blood oxygen level‐dependent (BOLD) signal between the patient (prior to rTMS, consistent with visual system status at time of DTI acquisition) and controls (Rafique et al., 2016). Probabilistic tractography was therefore employed to reconstruct intrahemispheric tracts seeded from the visual cortex (including primary and association cortices, i.e., Brodmann areas 17, 18, 19), to an end point in subregions of frontal lobe gyri (superior, middle, and inferior), temporal lobe gyri (superior and middle temporo‐occipital), parietal lobules (superior and inferior), and precentral and postcentral gyri, independently. In addition, we reconstructed the optic radiations using a seed at the lateral geniculate body ending at the primary visual cortex. A converse reconstruction of these intrahemispheric tracts was performed using a seed at the primary visual cortex ending at the lateral geniculate body as these are shown to differ from feedforward connections (Shipp, 2007; Sillito, Cudeiro, & Jones, 2006). Tracts were reconstructed for all participants and both hemispheres. We further investigated interhemispheric visual cortex connectivity by generating tracts between visual cortices in both directions, that is, tracts seeded from the right visual cortex ending in the left visual cortex, and vice versa. Reference to seed and end points does not imply tract directionality, but strictly refers to the generation of tracts from every point in the seed to only enter and terminate at the target end point. Probabilistic tractography was determined in standard MNI space. Tracts were visually inspected for changes in size and orientation compared with the contralateral hemisphere, and comparing the patient with controls. Complete details on reconstructing probabilistic fiber tractography are provided in Supporting Information.

## 3 RESULTS

### 3.1 Diffusion tensor indices

A color‐coded FA map for the patient indicating the lesion site is shown in Figure 1b. Table 1 contains values for diffusion tensor indices obtained from the major white matter tracts and demonstrates significant differences in diffusivity between the patient and controls.

### 3.2 Probabilistic fiber tractography

**Figure 2:**
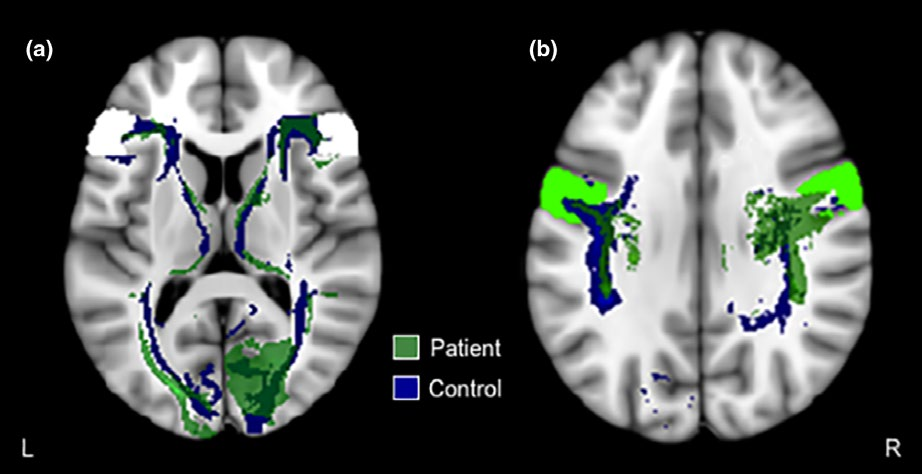
Probabilistic fiber tractography for intrahemispheric tracts seeded from the visual cortex to (a) inferior frontal gyrus (white mask), and (b) precentral gyrus (lime green mask). Only one control example is presented here, all other controls are presented in Supporting Information Figures S1–S2, respectively. See Figure 5 for an example of the visual cortex mask (red mask). L: left (contralesional in the patient); R: right (ipsilesional in the patient)

Reconstruction of intrahemispheric tracts seeded from the visual cortex to the inferior frontal gyrus showed an increase in tracts for the patient within the ipsilesional visual cortex compared with the contralesional hemisphere and controls (Figure 2a). Similar to that, for intrahemispheric tracts seeded from the ipsilesional visual cortex to the precentral gyrus, the patient showed increased tracts close to the precentral gyrus relative to the contralesional hemisphere and controls (Figure 2b).

**Figure 3:**
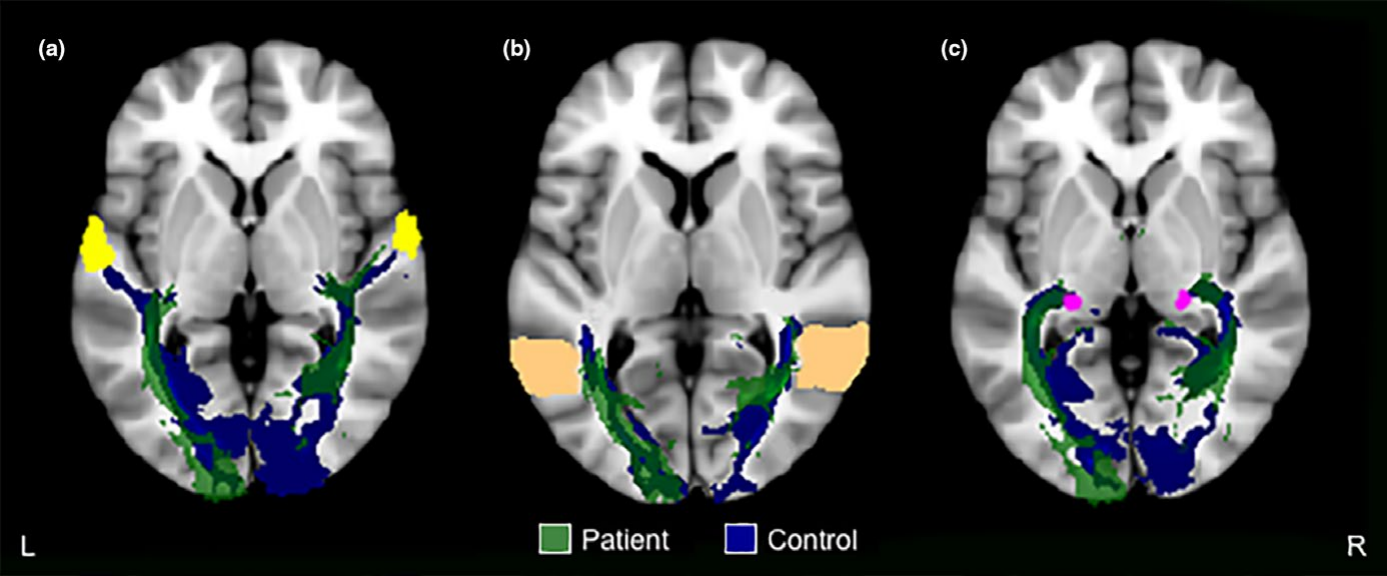
Probabilistic fiber tractography for intrahemispheric tracts seeded from the visual cortex to (a) superior temporal gyrus (yellow mask), (b) middle temporo‐occipital gyrus (peach mask), and (c) lateral geniculate body (pink mask). Only one control example is presented here, all other controls are presented in Supporting Information Figures S3–S5. See Figure 5 for an example of the visual cortex mask (red mask). L: left (contralesional in the patient); R: right (ipsilesional in the patient)

Probabilistic tractography for the patient further showed incomplete intrahemispheric tracts seeded from the ipsilesional visual cortex to superior temporal and middle temporo‐occipital gyri, and the lateral geniculate body, compared with the contralesional hemisphere and controls (Figure 3a–c, respectively). This is observed from the absence of intrahemispheric tracts within the ipsilesional visual cortex in the patient; instead, the tracts originate further anteriorly, closer to anterior temporo‐occipital regions.

**Figure 4:**
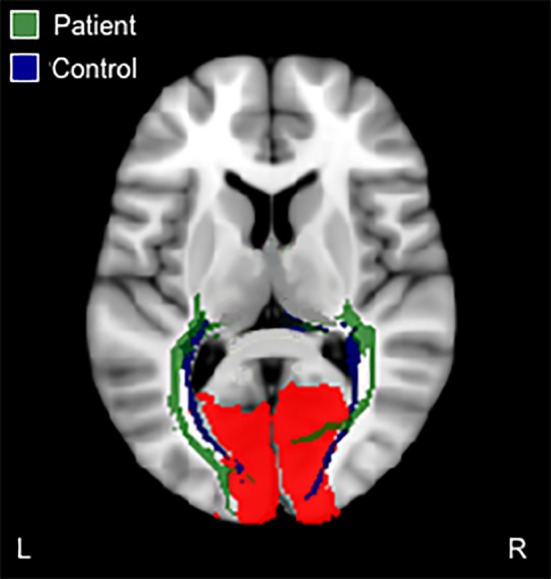
Probabilistic fiber tractography of the optic radiations. Tracts were seeded from the lateral geniculate body to the visual cortex (red mask). Only one control example is presented here, all other controls are presented in Supporting Information Figure S6. See Figure 3c for an example of the lateral geniculate body mask (pink mask). L: left (contralesional in the patient); R: right (ipsilesional in the patient)

Reconstruction of the optic radiations in the patient shows a marked displacement of ipsilesional optic radiations relative to the contralesional hemisphere and controls. The terminal fibers of the patient’s ipsilesional optic radiations are seen to deviate anterior to the lesion site (Figure 4).

**Figure 5:**
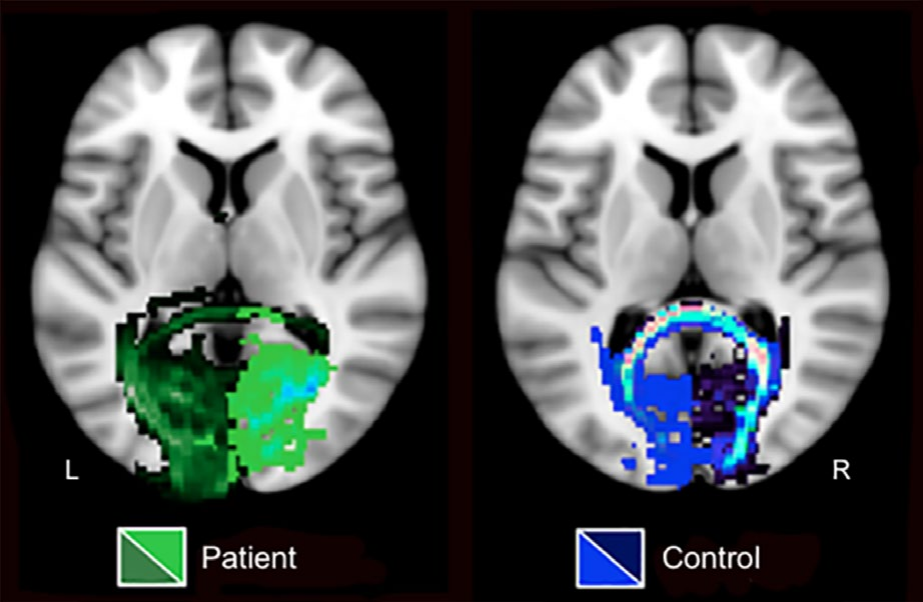
Probabilistic fiber tractography of interhemispheric connections between visual cortices. In the patient, tracts seeded from the ipsilesional (R) hemisphere (light green tracts) do not cross the midline to terminate in the contralesional (L) hemisphere, while contralesional to ipsilesional (dark green tracts) interhemispheric tracts do. In controls, tracts seeded from the right visual cortex (dark blue tracts) do cross the midline to connect with the left visual cortex, and vice versa (blue tracts), resulting in overlapping interhemispheric connections (light blue and pink tracts). Only one control example is presented here, all other controls are presented in Supporting Information Figure S7. L: left (contralesional in the patient); R: right (ipsilesional in the patient)

Altered interhemispheric communication between visual cortices is observed in the patient. Tracts seeded from the ipsilesional visual cortex fail to terminate in the contralesional visual cortex, and instead discontinue at the midline. However, the converse is spared as tracts seeded from the contralesional visual cortex continue past the midline and terminate in the ipsilesional visual cortex (Figure 5). In comparison, control participants demonstrated overlapping interhemispheric connections (Figure 5).

Given that directionality cannot be inferred from probabilistic tractography, it is important to note that tracts seeded from the lateral geniculate body to the visual cortex are intermingled with and cannot be differentiated from the converse reconstruction of these tracts (i.e., seeded from the visual cortex to the lateral geniculate body) using this method. The same applies to interhemispheric visual cortex tracts.

For ease of comparison of probabilistic fiber tractography between the patient and controls, we only present data of one control example here. The results of probabilistic tractography for all other controls are presented in Supporting Information Figures S1–S7. Despite interindividual variation in tracts, the patient consistently showed these differences compared with all control participants.

No differences were observed between the patient and controls for intrahemispheric tracts seeded from the visual cortex to superior and middle frontal gyri, and postcentral gyrus. Probabilistic tractography did not reveal tracts from the visual cortex to the middle temporal gyrus or parietal lobules in the patient or controls.

## 4 DISCUSSION

Diffusion imaging highlights changes in diffusion indices of major white matter tracts associated with the patient’s lesion site compared with controls. These differences are consistent with regions previously implicated in visual hallucinations. Moreover, probabilistic fiber tractography demonstrates changes in white matter tracts between the visual cortex and remote regions in the patient that are consistent with previously observed alterations in functional activation between the patient and controls.

### 4.1 White matter changes with occipital stroke and associated vision loss

In the patient, we observed incomplete intrahemispheric tracts from the ipsilesional visual cortex to superior temporal and middle temporo‐occipital gyri (Figure 3a–b, respectively). This finding is consistent with observed differences in functional activation within these regions between the patient and controls during a basic visual task (Rafique et al., 2016). Although there was only an increase in intrahemispheric tracts from the ipsilesional visual cortex to the inferior frontal gyrus (Figure 2a), the patient has shown significantly greater functional activation in bilateral inferior frontal gyri compared with controls (Rafique et al., 2016). These regions are connected via major white matter tracts, the superior longitudinal and inferior fronto‐occipital fasciculi. The superior longitudinal fasciculus connects frontal, parietal, temporal, and occipital lobes (Catani, Howard, Pajevic, & Jones, 2002), while the inferior fronto‐occipital fasciculus connects the frontal lobe with posterior aspects of parietal, temporal, and occipital lobes (Martino, Brogna, Robles, Vergani, & Duffau, 2010). The inferior fronto‐occipital fasciculus further passes through the lateral geniculate body and claustrum (Kier, Staib, Davis, & Bronen, 2004). Not only did the patient show incomplete tracts from the ipsilesional visual cortex to the intrahemispheric lateral geniculate body (Figure 3c), our previous fMRI findings implicate the ipsilesional claustrum in the genesis of visual hallucinations in this patient (Rafique et al., 2016). The inferior longitudinal fasciculus connects anterior temporal and posterior occipito‐temporal regions of the occipital lobe, including visual association areas, fusiform, and parahippocampal gyri (Catani, Jones, & Donato, 2003; Wakana, Jiang, Nagae‐Poetscher, Van Zijl, & Mori, 2004). Given that the lesion site involved right visual association areas (Figure 1; Rafique et al., 2016), we expect the right inferior longitudinal fasciculus to be compromised (Figure 1; Table 1). The visual association areas (e.g., lingual gyrus and cuneus) showed the most obvious alterations in functional activity in our patient and were considered the primary regions contributing to the perception of visual hallucinations (Rafique et al., 2016).

Changes in FA and/or AD of the superior (including temporal aspects) and inferior longitudinal fasciculi in the patient (Table 1) are therefore not surprising given the disruption to diffusivity at the stroke site (Figure 1), and consequent changes in tractography between regions concerned with these fasciculi (Figures 2, 3). Altered FA suggests changes to white matter microstructure (Jones, Simmons, Williams, & Horsfield, 1999) indicative of change in aspects of connectivity (Jones, Knösche, & Turner, 2013). AD measures are considered variable in white matter changes and pathology; however, a decrease is observed in axonal injury/damage (Song et al., 2002). Despite involvement of intrahemispheric ipsilesional tracts in the patient (Figures 2a and 3b–c) implicating the ipsilesional inferior fronto‐occipital fasciculus, the contralesional inferior fronto‐occipital fasciculus showed significantly lower AD (Table 1). A possible explanation for contralesional inferior fronto‐occipital fasciculus involvement may be from sharing of axons implicated with the lesion site.

Alterations in interhemispheric white matter connectivity between visual cortices in the patient (Figure 5) offer further explanation to the disorganized and imbalanced functional activity in visual processing regions previously observed across both hemispheres, for example, greater functional activity in the contralesional hemisphere (Rafique et al., 2016).

### 4.2 Compensatory white matter changes with occipital stroke and associated vision loss

The marked displacement of the ipsilesional optic radiations in the patient (Figure 4) suggests recovery of function with new connections via recruitment of peri‐infarct regions that adapt to new roles after stroke (Carmichael, 2003; Cramer, 2008). Damage to the optic radiation pathway is consistent with the presence of hemianopia visual field loss in the patient (Rafique et al., 2016). However, we do not see this recovery in the converse reconstruction of these tracts (seeded from the visual cortex to the lateral geniculate body) in the patient (Figure 3c). It is quite notable that, the location of terminal fibers of the ipsilesional optic radiations is consistent with the location of disorganized functional activity anterior to the patient’s lesion at the lingual gyrus (Rafique et al., 2016). In this patient, it appears that input from the retina to ipsilesional visual cortex via reorganization of optic radiations produces sustained sensory responses in new structural circuits in peri‐infarct regions; and this aberrant neuronal firing produces the perception of phosphene hallucinations in the area of hemianopic visual field loss. Indeed, in vivo cortical recordings of mice show an effect of prolonged depolarization at remapped peri‐infarct regions and in regions with greater connectivity poststroke (Brown, Aminoltejari, Erb, Winship, & Murphy, 2009). A DTI study in an infant with perinatal temporo‐parieto‐occipital stroke shows the same pattern of regeneration of ipsilesional optic radiations (Seghier et al., 2005) as our patient. In both cases, the location of terminal fibers of the regenerated optic radiations is consistent with the location of disorganized ipsilesional functional activity. Moreover, ipsilesional functional activation in the infant was localized anterior to the lesion at the lingual gyrus in the same manner as our patient. No follow‐up was reported in the infant past 20 months of age; therefore, we cannot ascertain whether visual hallucinations manifested in the infant.

Lower FA and increased RD were observed in the contralesional corticospinal tract of the patient (Table 1). An increase in RD suggests white matter pathology (Song et al., 2002) in this motor pathway. Although motor deficits were not a consequence of stroke in our patient, corticospinal tract plasticity following early vision loss is thought to arise from overcoming difficulty with eye‐hand coordination from loss of vision (Yu et al., 2007). Many corticospinal neurons originate in the precentral gyrus (Dum & Strick, 1991), and accordingly, we observed an increase in tracts from the ipsilesional visual cortex to the intrahemispheric precentral gyrus (Figure 2b). These combined findings may compensate for the need for increased left eye‐hand coordination following left homonymous hemianopia visual field loss in the patient (Rafique et al., 2016). Consistent with these structural white matter changes, subtraction analysis of fMRI responses during a basic visual task between the patient and controls has shown greater patient activation in the ipsilesional (right) precentral gyrus, while controls showed greater activation in the left precentral gyrus (Rafique et al., 2016).

Changes in white matter tracts in the patient at both ipsi‐ and contralesional hemispheres stem from altered intra‐ and interhemispheric communication, for example, major forceps, or commissural tracts. Early lesions studies of the occipital lobe in animals show retinotopic reorganization and changes of neuronal response properties in the ipsilesional hemisphere, and aberrant distribution of callosal connections in both ipsi‐ and contralesional hemispheres (Payne & Lomber, 2001; Restrepo, Manger, Spenger, & Innocenti, 2003). Changes in contralesional tracts (Figure 2) may occur to compensate for changes in ipsilesional tracts, or from pruning that occurs from loss of interhemispheric communication following stroke (Grefkes et al., 2008). Increased ipsilesional tracts in the patient (Figure 2a) suggest recruitment of redundant/association neurons. Global overconnectivity is observed in patients with hemianopia following occipital stroke, with greater node strength in ipsilesional temporal and orbitofrontal areas as well as the contralesional visual association cortex (Guo, Jin, Feng, & Tong, 2014).

### 4.3 White matter changes associated with persistent visual hallucinations

Reduced FA of the contralesional anterior thalamic radiation in the patient (Table 1), which connects the anterior and dorsomedial thalamic nuclei with the prefrontal cortex (Wakana et al., 2004), suggests changes to white matter microstructure and connectivity of this tract (Jones et al., 1999, 2013). Thalamic degeneration is previously implicated in visual hallucinations (Manford & Andermann, 1998), consistent with its involvement in this patient.

The inferior longitudinal and inferior fronto‐occipital fasciculi are involved in visual perception (ffytche, 2008) and visual processing (Fox, Iaria, & Barton, 2008). As in our patient, visual hallucinations have been linked to lower FA in the inferior longitudinal fasciculus in individuals with schizophrenia (Ashtari et al., 2007), and dementia with Lewy bodies (Kantarci et al., 2010), suggesting an association of visual hallucinations with temporo‐occipital projections. Furthermore, frontal lesions can produce visual hallucinations in the temporal and occipital lobes thought to arise through the spread of increased activity from one area to another (Schneider, Crosby, Bagchi, & Calhoun, 1961). Consistent with this notion, direct electrical stimulation of the inferior frontal gyrus evokes visual hallucinations of faces considered to originate from activity propagated in the prefrontal cortex along white matter pathways to face processing regions (Vignal, Chauvel, & Halgren, 2000), for example, via the inferior longitudinal fasciculus. In addition to frontal stimulation, stimulation of the temporal lobe (Lee, Hong, Seo, Tae, & Hong, 2000; Penfield & Perot, 1963) and visual cortices (Lee et al., 2000) also evokes visual hallucinations. This suggests that it is not simply an increase and/or disorganization of activity in visual associated cortices that produce visual hallucinations (ffytche et al., 1998; Rafique et al., 2016), but rather the additional involvement of these white matter tracts (and accordingly specific networks) that differentiates these patients from those with similar disorders who do not experience visual hallucinations.

### 4.4 Limitations

Given the single case study approach of the present research, we appreciate that the data may be insufficient to generalize specific plasticity effects following occipital stroke associated with chronic visual hallucinations. In addition, due to the number of independent *t* tests, and the less conservative threshold of trending results (*p *<* *0.1), some differences in diffusion tensor indices between the patient and controls (Table 1) may reflect statistical noise. Further diffusion imaging studies in patients with visual pathway damage who perceive visual hallucinations and those who do not are necessary in order to isolate factors predisposing individuals to visual hallucinations. To our knowledge, other than the case of perinatal stroke (Seghier et al., 2005), there are no other studies reported in the literature investigating white matter structure in visual pathway damage with or without visual hallucinations. Knowledge of white matter structure is pivotal in elucidating underlying mechanisms of brain function associated with chronic and disruptive visual hallucinations in these patients. Evaluation of white matter connectivity in other leading causes of vision loss associated with visual hallucinations (e.g., age‐related macular degeneration) is also needed to confirm or refute our hypotheses implicating specific structural networks and remapping with visual hallucinations.

The patient presents with a benign subarachnoid cyst at the occipital pole. The cyst impinges on deeper occipital and cerebellar structures, and its location is sufficiently far removed that we would not expect it to directly affect the reconstruction of white matter tracts in this study. Therefore, the cyst would not account for the atypical anatomy of these tracts. Our results continue to show clear disturbance to white matter tracts in regions that are far from the cyst (e.g., Figure 2a). Our tractography findings in the patient may also be influenced by diffusion properties of the lesion, for example, diffusion values within the lesion may be outside of probabilistic tractography thresholds. A greater number of streamlines (tracts) can occur in probabilistic tractography in cases of Wallerian degeneration, where intact pathways with higher anisotropy will show a greater number of streamlines than the damaged pathways (Jones, 2010). However, the probabilistic tractography approaches used in this study (multifiber and a priori regions of interest) are shown to track reliably (Behrens, Berg, Jbabdi, Rushworth, & Woolrich, 2007; Dyrby et al., 2007), are fairly accurate in cases of crossing fibers, and can track through areas of low FA (Jones, 2010) such as degeneration of microstructure following stroke and in artefacts (Behrens et al., 2003; Jones, Travis, Eden, Pierpaoli, & Basser, 2005). As we are unable to acquire longitudinal data, we can only speculate as to the nature of the tracts with respect to regeneration. Further, changes in diffusion tensor indices in our patient cannot simply be interpreted as compromised microstructure of white matter, for example, specific to axonal changes or neurodegeneration. Interpretation of diffusion indices is uncertain when other factors such as glial cells or crossing fibers of similar density may contribute to water diffusion (Jbabdi, Behrens, & Smith, 2010). We can be more certain that axonal loss has occurred from known Wallerian degeneration in stroke, and accordingly the degeneration of neurons.

Although we are cognizant that this is a single case study and acknowledge that this case report using DTI is speculative, we have attempted to constrain the methods to the best of our ability by comparing to an age‐matched control group, as well as intra‐individual tracts, and previous fMRI data to strengthen the validity of the current findings.

## 5 CONCLUSION

In short, our results point toward interruption of specific white matter tracts (networks) and cortical remapping as the root of disorganized functional activity in the patient that leads to the continued perception of visual hallucinations. Cortical reorganization may offer explanation for the chronic and persistent nature of the hallucinations in this patient, differentiating this case from the more common reports of acute visual hallucinations (Manford & Andermann, 1998; Vaphiades et al., 1996). Diffusion imaging and fiber tractography combined with fMRI in future studies will provide a more accurate visualization of plasticity following visual pathway damage associated with visual hallucinations in order to ascertain treatment modalities directed at improving functional outcome.

## ACKNOWLEDGMENTS

We would like to thank all participants for their contribution.

## CONFLICT OF INTERESTS

None declared.
