# Supplementary material for: Altered white matter connectivity associated with visual hallucinations following occipital stroke
Source: Brain Behav. 2018 May 21;8(6):e01010. doi: 10.1002/brb3.1010 (PMC5991596; doi:10.1002/brb3.1010)
Supplement: Supplementary file 1 [file BRB3-8-e01010-s001.pdf]

SUPPORTING INFORMATION

Altered white matter connectivity associated with visual hallucinations following occipital stroke

Sara A. Rafique[1], John R. Richards[2], and Jennifer K. E. Steeves[1,a]

[1]Centre for Vision Research and Department of Psychology, York University, Toronto, Canada
[2]Department of Emergency Medicine, University of California, Davis, Medical Center, Sacramento, CA, USA

[a]Corresponding author email: steeves@yorku.ca

SUPPLEMENTARY METHODS

Magnetic resonance imaging acquisition

Diffusion tensor imaging (DTI) was obtained with whole-brain diffusion-weighted scanning using 2-dimensional gradient echo echo-planar sequencing (number of contiguous axial slices = 56; in-plane resolution = 1.5 x 1.5 mm; slice thickness = 2 mm with no interslice gap; imaging matrix = 128 x 128; repetition time (TR) = 6900 ms; echo time (TE) = 86 ms; field of view (FoV) = 192 mm) with 64 isotropically distributed orientations for the diffusion-sensitising gradients at a *b*-value of 1000 s/mm^2, and b = 0 s/mm^2 for T2-weighted images.

Structural images were acquired with a T1-weighted sequence using three-dimensional magnetisation-prepared rapid gradient-echo (MPRAGE) (number of slices = 192; in-plane resolution = 1 x 1 mm; slice thickness = 1 mm; imaging matrix = 256 x 256; TR = 1900 ms; TE = 2.5 ms; inversion time (TI) = 900 ms; flip angle = 9°; FoV = 256 mm).

A T2-weighted turbo spin echo fluid attenuated inversion recovery (FLAIR) imaging sequence was obtained in the patient to localise the lesion site (number of slices = 25; in-plane resolution = 0.9 x 0.9 mm; slice thickness = 4 mm; imaging matrix = 256 x 256; TR = 900 ms; TE = 93 ms; TI = 2500 ms; flip angle = 130°; FoV = 220 mm).

Data analyses

All DTI analyses were performed using FSL's DTI processing pipeline (FMRIB, Oxford, UK; www.fmrib.ox.ac.uk/fsl).

Image processing and normalisation

Diffusion images were corrected for eddy current induced spatial distortions and head motion using affine registration to the reference (*b*0) volume[1] within the FDT toolbox, and brain-extracted using BET to remove skull and head tissue.[2] T1-weighted images also underwent brain-extraction using BET. Diffusion tensor models were fitted at each voxel of the eddy current corrected diffusion data using standard linear regression with DTIFIT of the FDT toolbox. The brain-extracted T1-weighted images underwent linear registration to diffusion space, and also to standard MNI space (MNI152, 1x1x1 mm) using FLIRT[3] default parameters (affine 12 degrees of freedom; correlation ratio cost function; tri-linear interpolation). Diffusion images were non-linearly registered to T1-weighted structural and standard MNI spaces (MNI152, 1x1x1 mm) to derive transformation matrices using FNIRT registration of FDT[4] and default parameters (normal search; correlation ratio cost function; 6 and 12 degrees of freedom for structural and standard space, respectively).

Diffusion tensor indices

Tract-Based Spatial Statistics (TBSS) was used to obtain the diffusion tensor indices fractional anisotropy (FA), mean diffusivity (MD), axial diffusivity (AD), and radial diffusivity (RD) from major white matter tracts. AD was defined as the

principal diffusion eigenvalue (λ1), and RD as the mean of the second and third eigenvalues (λ2 + λ3/2). Using the diffusion-weighted brain images computed from DTIFIT, each participant's FA image underwent preprocessing to remove outliers from the diffusion tensor fitting. The FA image was then aligned to standard MNI space using affine non-linear registration, and thinned to create a binary skeleton image using a threshold of FA > 0.2. To obtain the non-FA diffusion indices, the FA-derived non-linear transformations were applied to the MD, AD, and RD images, and projected onto the FA binary skeleton to estimate projection vectors from the same voxels as in the FA analysis. These steps were repeated for all participants separately.

The major white matter tracts were obtained from John Hopkins University (JHU) White-Matter Tractography Atlas provided with FSL.[6] Tract threshold probability was set to 15% to allow for inter-subject variation and tracts were binarised. Each binarised tract was coregistered (masked) to each participant's binary skeleton diffusion image using *fslmaths*. The fit of the white matters tracts were manually assessed to ensure accurate overlap of tracts and skeleton images. Tract values were then extracted from skeletonised diffusion images for all diffusion tensor indices for each participant.

Probabilistic fibre tractography

To reconstruct tracts of interest we used FDT's BEDPOSTX, which uses Markov Chain Monte Carlo sampling to estimate diffusion parameters at each voxel, to model crossing fibres within each voxel of the corrected diffusion data.[7] We then used PROBTRACKX within the FDT toolbox to generate probabilistic tractography including crossing fibres[7] in MNI space. A two-region of interest approach was used to isolate streamlines (tracts). Fibre tracking involved 5000 streamlines drawn from each voxel in the seed mask through the probability distribution on principle fibre direction, and ended at a pathway curvature threshold of 0.2 (~80°) and a maximum number of 2000 steps (step length of 0.5 mm) (default parameters).

A seed mask was placed at the visual cortex (to include primary and association cortices, i.e., Brodmann areas 17, 18, 19) to waypoint (identifies tracts passing through this point only) and terminate (end point) at the following regions: frontal lobe gyri (superior, middle, and inferior), temporal lobe gyri (superior, and middle temporo-occipital), parietal lobules (superior, and inferior), and precentral and postcentral gyri. To minimise false positive tracts, an exclusion mask consisting of cerebral white matter of the opposite hemisphere was used to limit tracts within the ipsilateral hemisphere.

We further reconstructed the optic radiations by placing a seed mask at the lateral geniculate body, and waypoint and termination masks at the primary visual cortex. Converse tracts were generated from the primary visual cortex (seed mask) to the lateral geniculate body (waypoint and termination masks). A cerebral white matter exclusion mask of the opposite hemisphere was used to limit tracts within the ipsilateral hemisphere

To investigate interhemispheric connectivity between the visual cortices, a seed mask was placed at one hemisphere of the visual cortex with waypoint and termination masks placed at the contralateral visual cortex (with no exclusion mask). This was reversed to generate interhemispheric connections between the visual cortices for the opposite direction.

SUPPLEMENTARY RESULTS

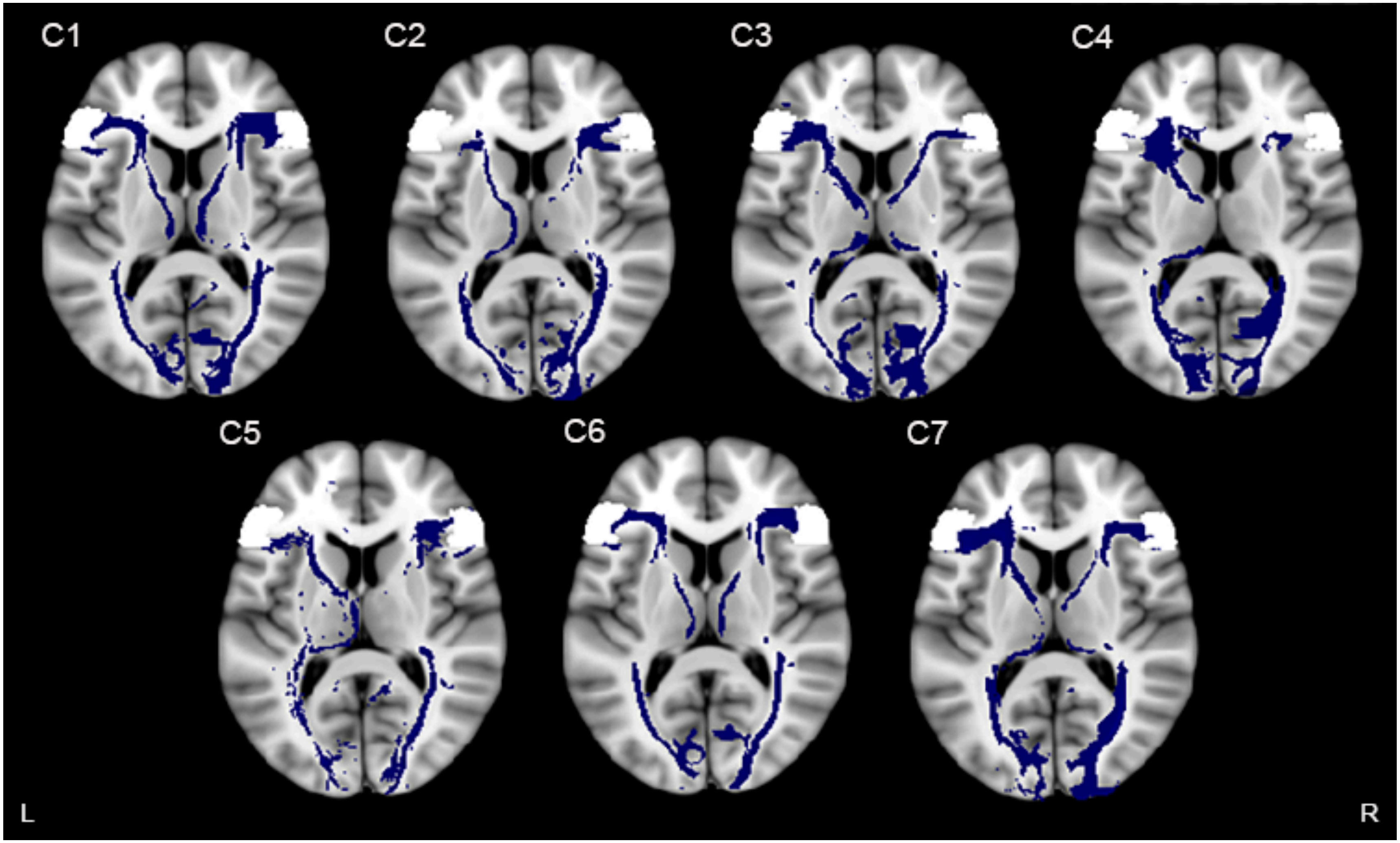

Figure S1. Probabilistic fibre tractography for intrahemispheric tracts seeded from the visual cortex to the inferior frontal gyrus (white mask) for all control participants (C1-C7). Results for C1 are also presented in the main article as a representative comparison to the patient's probabilistic fibre tractography. R = right; L = left.

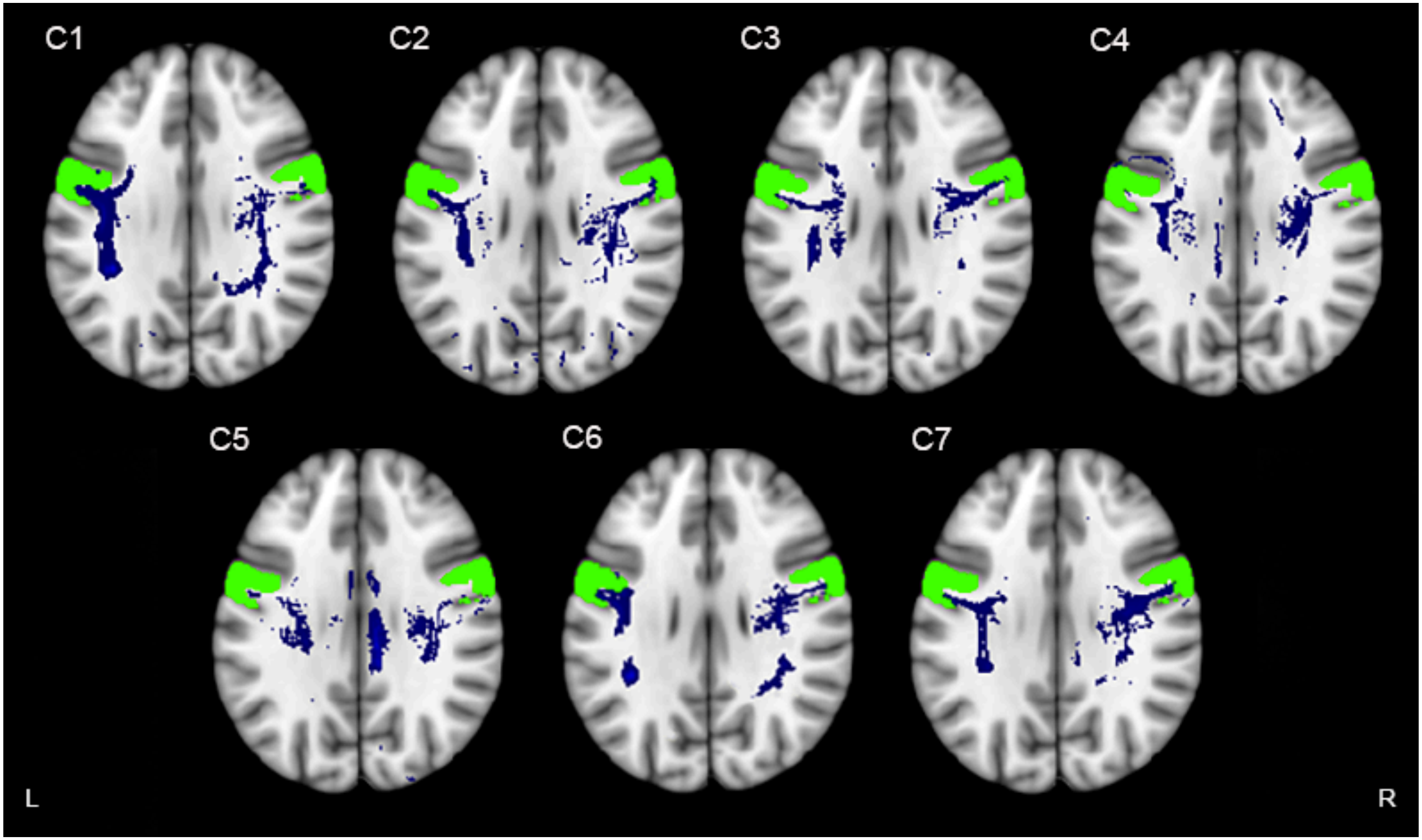

Figure S2. Probabilistic fibre tractography for intrahemispheric tracts seeded from the visual cortex to the precentral gyrus (lime green mask) for all control participants (C1-C7). Results for C1 are also presented in the main article as a representative comparison to the patient's probabilistic fibre tractography. R = right; L = left.

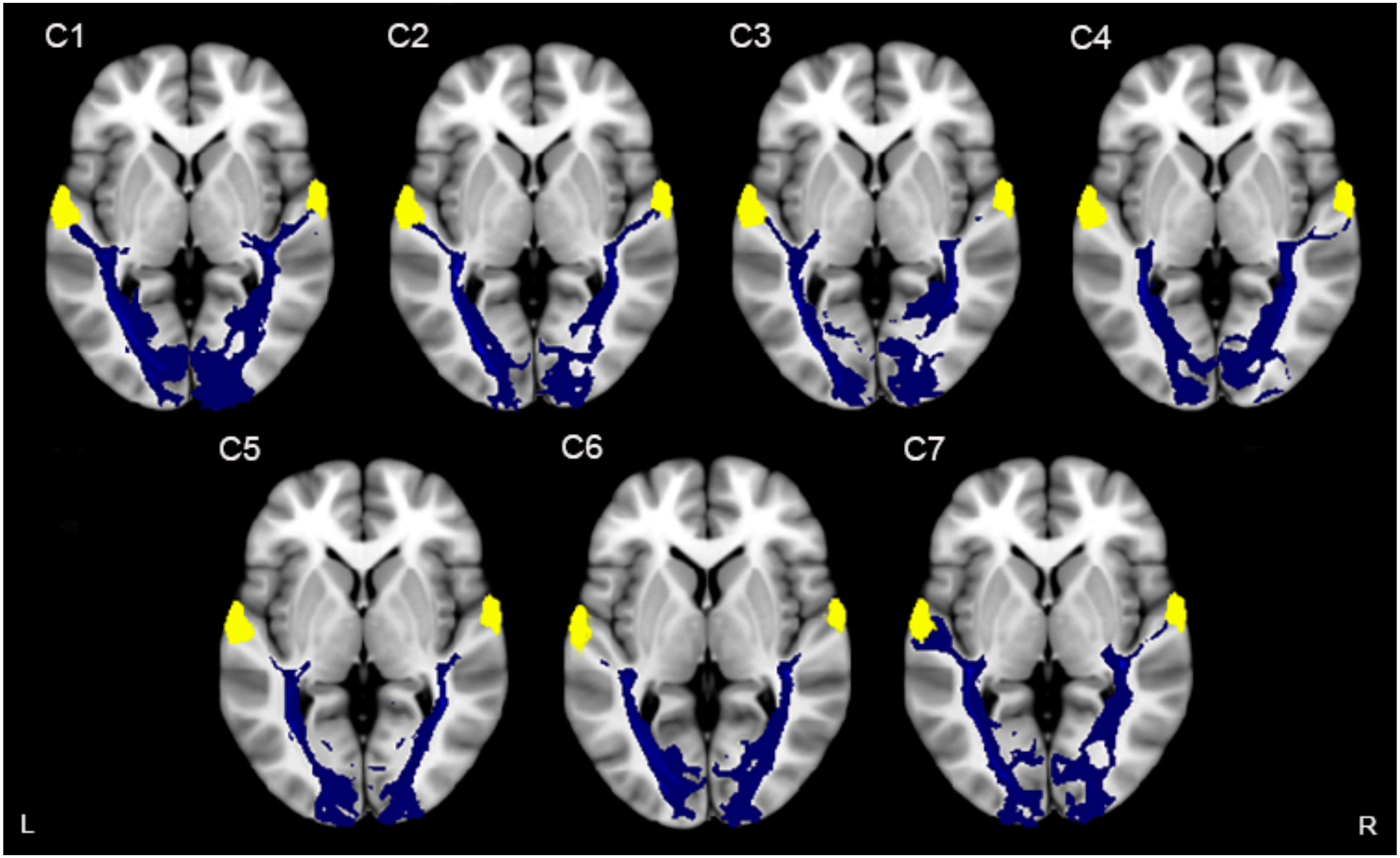

Figure S3. Probabilistic fibre tractography for intrahemispheric tracts seeded from the visual cortex to the superior temporal gyrus (yellow mask) for all control participants (C1-C7). Results for C1 are also presented in the main article as a representative comparison to the patient's probabilistic fibre tractography. R = right; L = left.

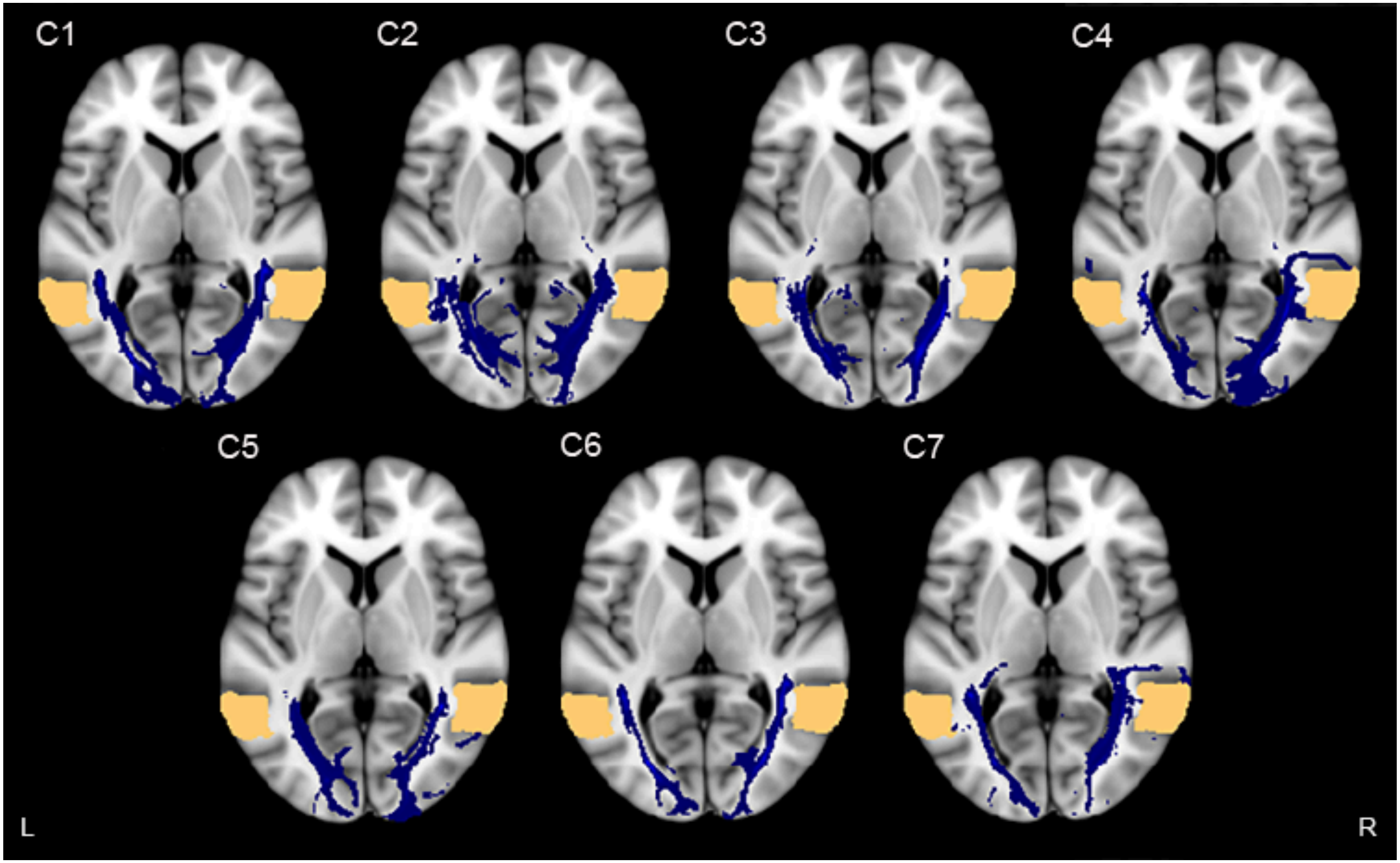

Figure S4. Probabilistic fibre tractography for intrahemispheric tracts seeded from the visual cortex to the middle temporo-occipital gyrus (peach mask) for all control participants (C1-C7). Results for C1 are also presented in the main article as a representative comparison to the patient's probabilistic fibre tractography. R = right; L = left.

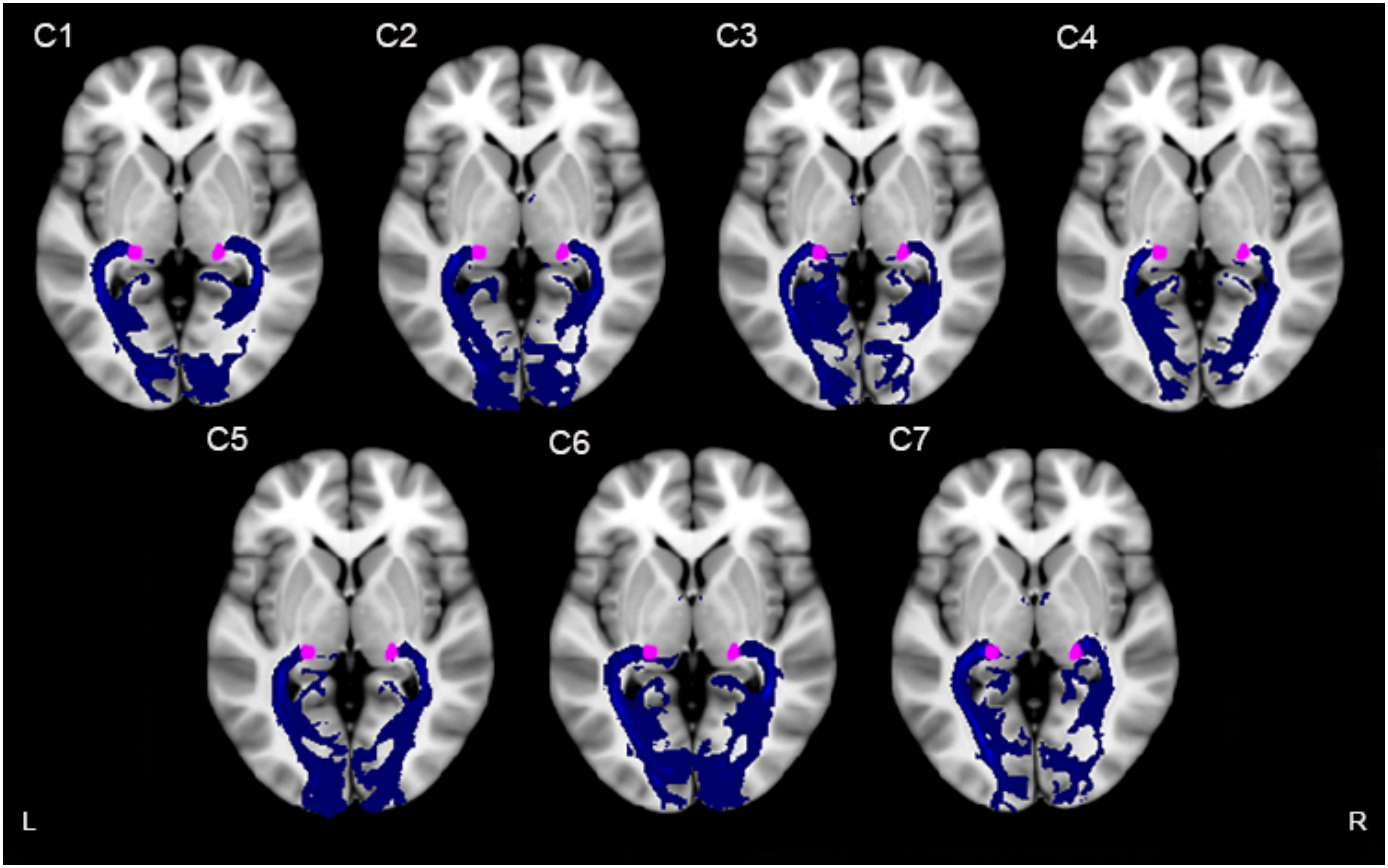

Figure S5. Probabilistic fibre tractography for intrahemispheric tracts seeded from the visual cortex to the lateral geniculate body (pink mask) for all control participants (C1-C7). Results for C1 are also presented in the main article as a representative comparison to the patient's probabilistic fibre tractography. R = right; L = left.

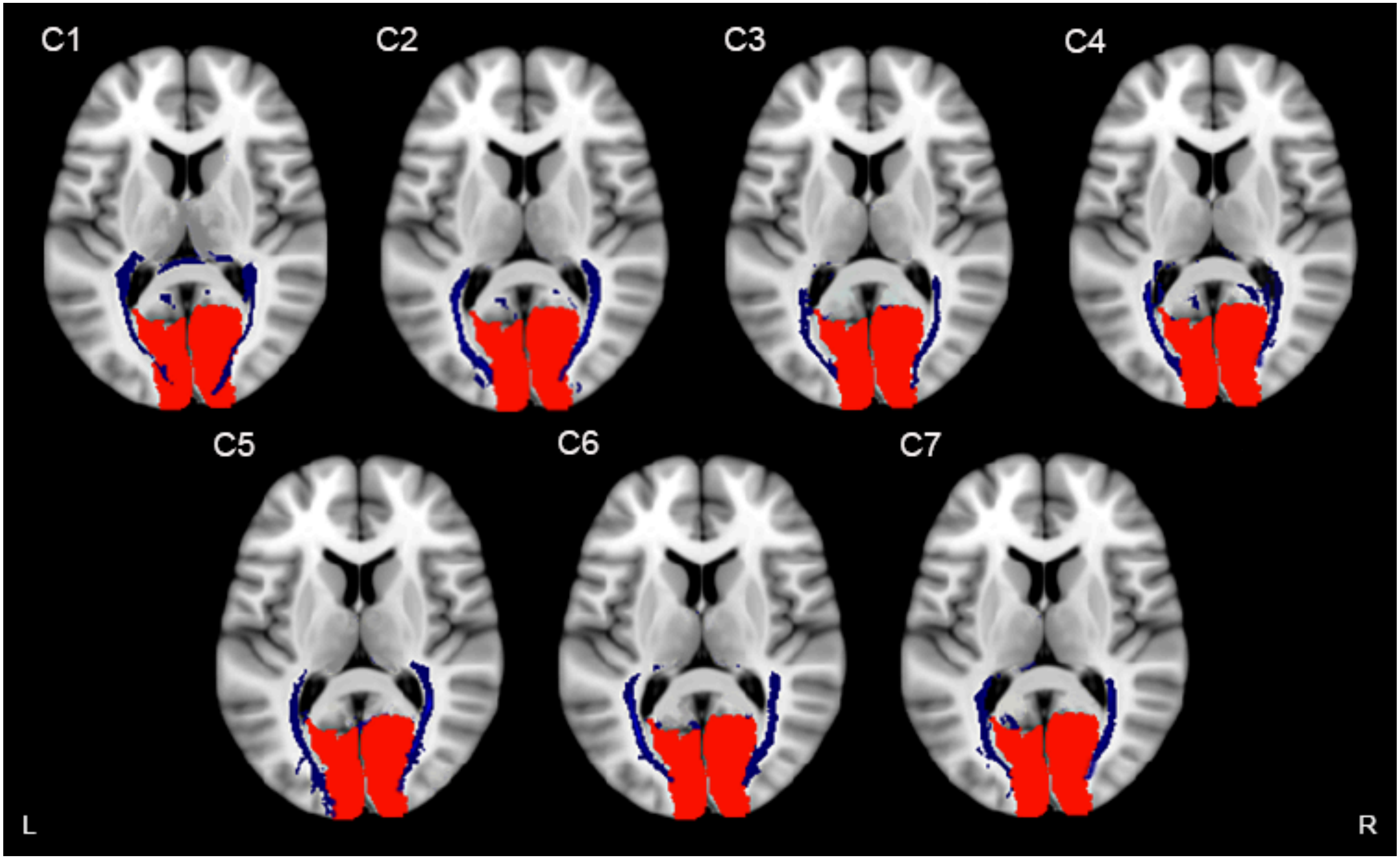

Figure S6. Probabilistic fibre tractography for intrahemispheric tracts seeded from the lateral geniculate body to the visual cortex (red mask) for all control participants (C1-C7). Results for C1 are also presented in the main article as a representative comparison to the patient's probabilistic fibre tractography. R = right; L = left.

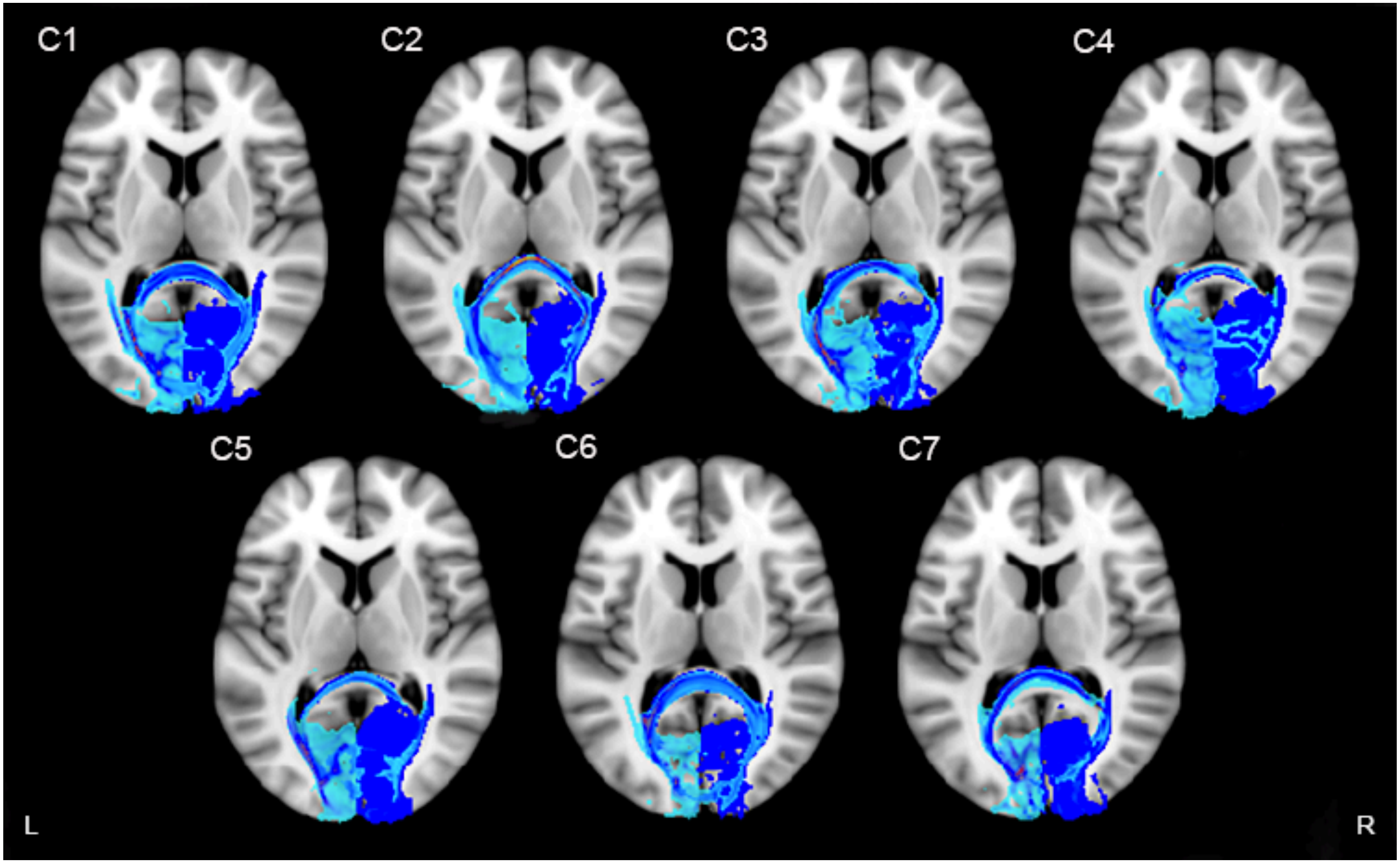

Figure S7. Probabilistic fibre tractography of interhemispheric tracts between visual cortices for all control participants (C1-C7). Results for C1 are also presented in the main article as a representative comparison to the patient's probabilistic fibre tractography. R = right; L = left.

SUPPLEMENTARY REFERENCES

1. Andersson JL, Sotiropoulos SN (2016): An integrated approach to correction for off-resonance effects and subject movement in diffusion MR imaging. Neuroimage 125:1063-1078.
2. Smith SM (2002): Fast robust automated brain extraction. Hum Brain Mapp 17:143-155.
3. Jenkinson M, Bannister P, Brady M, Smith S (2002): Improved optimization for the robust and accurate linear registration and motion correction of brain images. Neuroimage 17:825-841.
4. Andersson JL, Jenkinson M, Smith S (2007): Non-linear registration, aka Spatial normalisation. FMRIB technical report TR07JA2. FMRIB Analysis Group of the University of Oxford. Available at http://www.fmrib.ox.ac.uk/datasets/techrep/
5. Smith SM, Jenkinson M, Johansen-Berg H et al (2006): Tract-based spatial statistics: voxelwise analysis of multi-subject diffusion data. Neuroimage 31:1487-1505.
6. Wakana S, Caprihan A, Panzenboeck MM et al (2007): Reproducibility of quantitative tractography methods applied to cerebral white matter. Neuroimage 36:630-644.
7. Behrens TE, Berg HJ, Jbabdi S, Rushworth MF, Woolrich MW (2007): Probabilistic diffusion tractography with multiple fibre orientations: What can we gain? Neuroimage 34:144-155.
